# A polygenic *p* factor for major psychiatric disorders

**DOI:** 10.1038/s41398-018-0217-4

**Published:** 2018-10-02

**Authors:** Saskia Selzam, Jonathan R. I. Coleman, Avshalom Caspi, Terrie E. Moffitt, Robert Plomin

**Affiliations:** 1 MRC Social, Genetic and Developmental Psychiatry Centre, Institute of Psychiatry, Psychology and Neuroscience, King’s College London, London, UK; 2 NIHR Biomedical Research Centre for Mental Health, South London and Maudsley NHS Trust, London, UK; 3 Department of Psychology and Neuroscience, Duke University, Durham, USA; 4 Center for Genomic and Computational Biology, Duke University, Durham, USA; 5 Department of Psychiatry and Behavioral Sciences, Duke University Medical Center, Durham, USA

## Abstract

It has recently been proposed that a single dimension, called the *p* factor, can capture a person’s liability to mental disorder. Relevant to the *p* hypothesis, recent genetic research has found surprisingly high genetic correlations between pairs of psychiatric disorders. Here, for the first time, we compare genetic correlations from different methods and examine their support for a genetic *p* factor. We tested the hypothesis of a genetic *p* factor by applying principal component analysis to matrices of genetic correlations between major psychiatric disorders estimated by three methods—family study, genome-wide complex trait analysis, and linkage-disequilibrium score regression—and on a matrix of polygenic score correlations constructed for each individual in a UK-representative sample of 7 026 unrelated individuals. All disorders loaded positively on a first unrotated principal component, which accounted for 57, 43, 35, and 22% of the variance respectively for the four methods. Our results showed that all four methods provided strong support for a genetic *p* factor that represents the pinnacle of the hierarchical genetic architecture of psychopathology.

## Introduction

High comorbidity rates among psychiatric disorders^1^ have led to research investigating higher-order dimensions for psychopathology, including Internalizing (e.g., Anxiety and Depression), Externalizing (e.g., Hyperactivity and Conduct Disorder), and Psychotic Experiences (e.g., Schizophrenia and Bipolar Disorder)^2^. However, these higher-order dimensions also correlate with each other^3^, which suggests the possible existence of a general factor of psychopathology^4^. This general factor has been called the *p* factor^5^ as it captures the shared variance across psychiatric symptoms, and predicts a multitude of poor outcomes and general life impairment^6,7^.

Family studies support the hypothesis of a genetic *p* factor in that genetic influences on psychopathology appear to be general across disorders rather than specific to each disorder. For example, psychiatric disorders do not breed true—parental psychopathology predicts offspring psychiatric disorders but with little specificity^8^. Family research has found substantial genetic correlations between pairs of disorders, such as Major Depression and Generalized Anxiety Disorder^9^ and Schizophrenia and Bipolar Disorder^10^. Genetic overlap between internalizing and externalizing higher-order constructs has also been noted^11^, consistent with the hypothesis of a general *p* factor. The culmination of this research is a recent study of more than 3 million full and half-siblings using Swedish national register data that found evidence for a general genetic factor that pervades eight major psychiatric disorders as well as convictions for violent crimes^12^. Although genetic correlations were not presented, the average loading was 0.45 on a general genetic factor.

Genomic research also supports the hypothesis of a genetic *p* factor. The first hint came from genome-wide association (GWA) findings that single- nucleotide polymorphisms (SNPs) found to be associated with Schizophrenia were also associated with bipolar disorder^13^. In 2013, genetic correlations were first estimated from linear mixed model analyses (genome-wide complex trait analysis, GCTA) of individual genotype data for five psychiatric disorders in the Psychiatric Genomics Consortium (PGC)^14^. Schizophrenia, Bipolar Disorder and Major Depressive Disorder yielded the highest genetic intercorrelations (average = 0.53); the average genetic correlation among the five disorders, including Autistic Spectrum Disorder and Attention-Deficit/Hyperactivity Disorder, was 0.22.

Linkage-Disequilibrium Score Regression (LDSC)^15^ has made it possible to estimate genetic correlations from GWA summary statistics rather than requiring genotype data for individuals. This method is based on correlations in effect sizes across disorders taking into account linkage disequilibrium and the SNP heritabilities of the disorders. LDSC genetic correlations derived from summary GWA statistics for the same five PGC disorders are remarkably similar to the GCTA genetic correlations described above that used individual genotype data^16^. A recent LDSC analysis of eight psychiatric disorders again showed considerable correlations between Schizophrenia, Bipolar Disorder and Major Depressive Disorder (average = 0.41), and yielded an average genetic correlation of 0.21^17^, highlighting the relevance of testing the hypothesis of a genetic *p* factor.

Another approach that has not yet been systematically applied to test for a genetic *p* is to correlate genome-wide polygenic scores (GPS), although some GPS correlations between pairs of psychiatric disorders have been reported^18^. A GPS for a disorder is created for an individual by summing the count of alleles shown in GWA studies to be associated with the disorder, after weighting the alleles by the strength of their association^19^. The previously described PGC dataset was used to create polygenic scores for each of the five disorders^13^, and polygenic scores for Schizophrenia, Bipolar Disorder and Major Depressive Disorder predicted liability variance in the other disorders, again suggesting genetic overlap. However, as new GWA studies have been published since for Schizophrenia, Attention-Deficit/Hyperactivity Disorder and Autism Spectrum Disorder with considerably increased sample sizes, replication is needed. GPS correlations between disorders are related to genetic correlations, but differ from the genetic correlations estimated from other methods because they index both the relationship between individual-specific genetic effects for traits in the population and genetic effects derived from an independent analysis. Nonetheless, GPS correlations provide another opportunity to test the hypothesis of a genetic *p* factor.

Based on the overwhelming evidence that favors a general *p* factor, we test whether a general *p* factor also emerges from genomic data. In the present study, we bring together genetic correlations for major psychiatric disorders derived from four genetic methods (family, GCTA, LDSC and GPS). We applied principal component analysis to correlation matrices derived from these four methods and estimate the amount of genetic variance explained by a genetic *p* factor. For the GPS approach, we constructed GPS for eight psychiatric disorders for each individual in a sample of 7 026 unrelated individuals from the Twins Early Development Study (TEDS)^20^.

Our hypothesis was that a general genetic factor would emerge from factor analyses of correlations derived from each of the four genetic methods. We also investigated the extent to which all disorders load on this general factor and the magnitude of their loadings.

## Methods

### Sample

This study included 7 026 unrelated (i.e., one member per twin pair), genotyped individuals from TEDS, a longitudinal birth cohort that recruited over 1 5000 twin pairs between 1994–1996 who were born in England or Wales. Despite some attrition, the remaining cohort, as well as the genotyped subsample have been shown to represent the UK population^20,21^. Written informed consent was obtained from parents. Project approval was granted by King’s College London’s ethics committee for the Institute of Psychiatry, Psychology and Neuroscience (05.Q0706/228).

### GPS calculation and GPS correlations

To obtain individual-specific genetic measures for psychiatric traits, we created eight GPS in our independent sample of 7026 individuals based on publicly available GWA summary statistics from the PGC: Schizophrenia, Bipolar Disorder, Major Depressive Disorder, Autism Spectrum Disorder, Attention-Deficit/Hyperactivity Disorder, Obsessive-Compulsive Disorder, Anorexia Nervosa, Post-Traumatic Stress Disorder (Supplementary Table S1). Following quality control and imputation (see Supplementary Methods S1 for details), genotypic data included 515 100 genotyped or imputed SNPs (info = 1). To calculate polygenic scores, we used a Bayesian approach, *LDpred*^22^, which modifies the summary statistic coefficients based on information on linkage disequilibrium (LD) and a prior on the effect size of each SNP. The final GPS is obtained as the sum of the trait-increasing alleles (each variant coded as 0, 1 or 2), weighted by the posterior effect size estimates. For our analyses, we used a prior that assumes a fraction of causal markers of 1 (for more information, see Supplementary Methods S2). All polygenic scores were adjusted for the first ten principal components of the genotype data, and chip, batch and plate effects using the regression method. The resulting standardized residuals were used for subsequent analyses.

In the TEDS sample, we created polygenic scores for the eight psychopathology traits. These scores followed a normal distribution and were used to generate a correlation matrix for these eight polygenic scores for use in subsequent analyses.

### Genetic correlations based on LDSC

LDSC is a method used to estimate SNP-heritability (SNP−*h*^2^) based on GWA summary statistics only, and relies on the principle that the presence of LD in the study sample is correlated with the upward bias of GWA test statistics^15^. Cross-trait LDSC^16^ is an extension of this method and makes it possible to estimate the genetic relationship between two traits. For each SNP, this method establishes the covariance of the test statistics for trait *x* and trait *y*, and regresses this value on the LD score of that SNP (i.e., the sum of the squared correlations of the SNP with its surrounding SNPs), whereby the slope represents the genetic covariance. The genetic correlation is obtained by standardizing the covariance by the SNP-*h*^2^ for both traits $({r}_{g}={\mathrm{cov}}_{xy}/{\sqrt{{h}_{{x}^{2}}}{h}_{{y}^2}})$. We applied cross-trait LDSC analysis on the same eight PGC summary statistics used for polygenic score creation to generate a genetic correlation matrix for further analysis. (For univariate SNP-*h*^2^ results using LDSC, see Supplementary Table S2.)

### Genetic correlations based on GCTA

In addition to GPS and LDSC analysis, we also obtained genetic correlation matrices through cross-sample bivariate GCTA based on genome-wide relatedness maximum likelihood^23^. Unlike LDSC, which uses GWA summary statistics, bivariate GCTA requires individual-level genotype data of unrelated individuals to estimate genetic correlations, implementing linear mixed model analysis. Cross-sample GCTA is an extension to bivariate GCTA^24^ and makes it possible to calculate genetic correlation estimates without requiring overlapping phenotypic information between samples. Rather, it compares genetic similarity between individuals that have the same disease status (case, control) for different disorders. For example, if cases of one disorder are genetically more similar to cases of a different disorder than to the respective controls, a positive genetic correlation can be inferred. For this study, we used published cross-sample GCTA genetic correlations^14^, which included five psychiatric disorders: Schizophrenia, Bipolar Disorder, Major Depressive Disorder, Autism Spectrum Disorder, and Attention-Deficit/Hyperactivity Disorder. (For univariate SNP-*h*^2^ estimates, see Supplementary Table S3.)

### Genetic correlations based on family data

Finally, we used genetic correlations based on quantitative genetic analysis comparing 3 475 122 Swedish full-siblings and half-siblings, who are genetically similar 50 and 25%, respectively, for additive genetic effects. This family study represents a very different methodology as compared to the other methods. Rather than using direct estimates based on DNA differences, it uses indirect estimates based on the relative resemblance of full siblings and half siblings. Because this family study, the only one of its kind, is so different from the other methods, it is especially valuable to compare its genetic correlations to those from the other three methods. The genetic correlations were not included in the original publication^12^ but were kindly prepared and shared by the lead author, Erik Pettersson of the Karolinska Institute. The analysis included seven psychopathology traits (Schizophrenia, Bipolar Disorder, Attention-Deficit/Hyperactivity Disorder, Major Depressive Disorder, Anxiety, Alcohol use Disorder and Drug Abuse), as well as convictions for Violent Crimes. Schizoaffective disorder was redundant with Schizophrenia (genetic correlation = 0.99) and thus omitted here (Supplementary Figure S1).

### Statistical analyses

#### Principal component analysis

To test the hypothesis that a general genetic *p* factor emerges from the genetic relationships among psychopathology traits, we performed eigenvalue decomposition through principal component analysis (PCA), which aims to maximize variation of the first principal component^25^. We applied PCA to genetic correlation matrices derived from family analysis (8 × 8 matrix), GCTA (5 × 5 matrix), LDSC (8 × 8 matrix), and GPS (8 × 8 matrix) to estimate the loadings of each psychiatric trait on this component and the variance explained by the first principal component.

We also tested the statistical significance of the factor loadings, which represent correlations between the original standardized variables and the factors. By calculating the *t*-statistic of the correlation coefficients, we were able to derive empirical *p*-values based on the *t*-statistic distribution with *n*−2 degrees of freedom^26^. Significance testing was applied only to family and GPS loadings because we were unable to obtain degrees of freedom for GCTA and LDSC data, which is required for the calculation of *t*. All tests were two-tailed and a significance level of *α* = 0.05 was accepted as statistically significant. In addition to testing statistical significance, we calculated the proportion of factor loadings with a magnitude of ≥|0.30|. This value is a commonly used threshold in the factor analysis literature, as it indicates that the factor explains ~10% of the variance in the measure^27^, therefore substantially contributing to the factor.

The decision of how many components to retain for rotation was based on three criteria: (i) the Kaiser criterion^28^ of eigenvalue *λ* > 1; (ii) parallel analysis^29^, and (iii) scree plot inspection^30^ (for a more detailed description, see Supplementary Methods S3). To improve interpretability of the extracted components, we performed oblique rotation using the *Oblimin* method. We chose this approach, which permits factors to be correlated, because previous work using phenotypic data showed considerable associations between latent psychopathology dimensions^3,5^.

Analyses were performed in the open-source software R^31^, using the *hornpa*^32^ package to perform parallel analysis, the *psych*^33^ package to conduct PCA (using the ‘principal’ function), and the *GPArotation*^34^ package to apply oblique rotation. Analysis scripts are available from the first author upon request.

## Results

### Genetic correlations

**Fig. 1:**
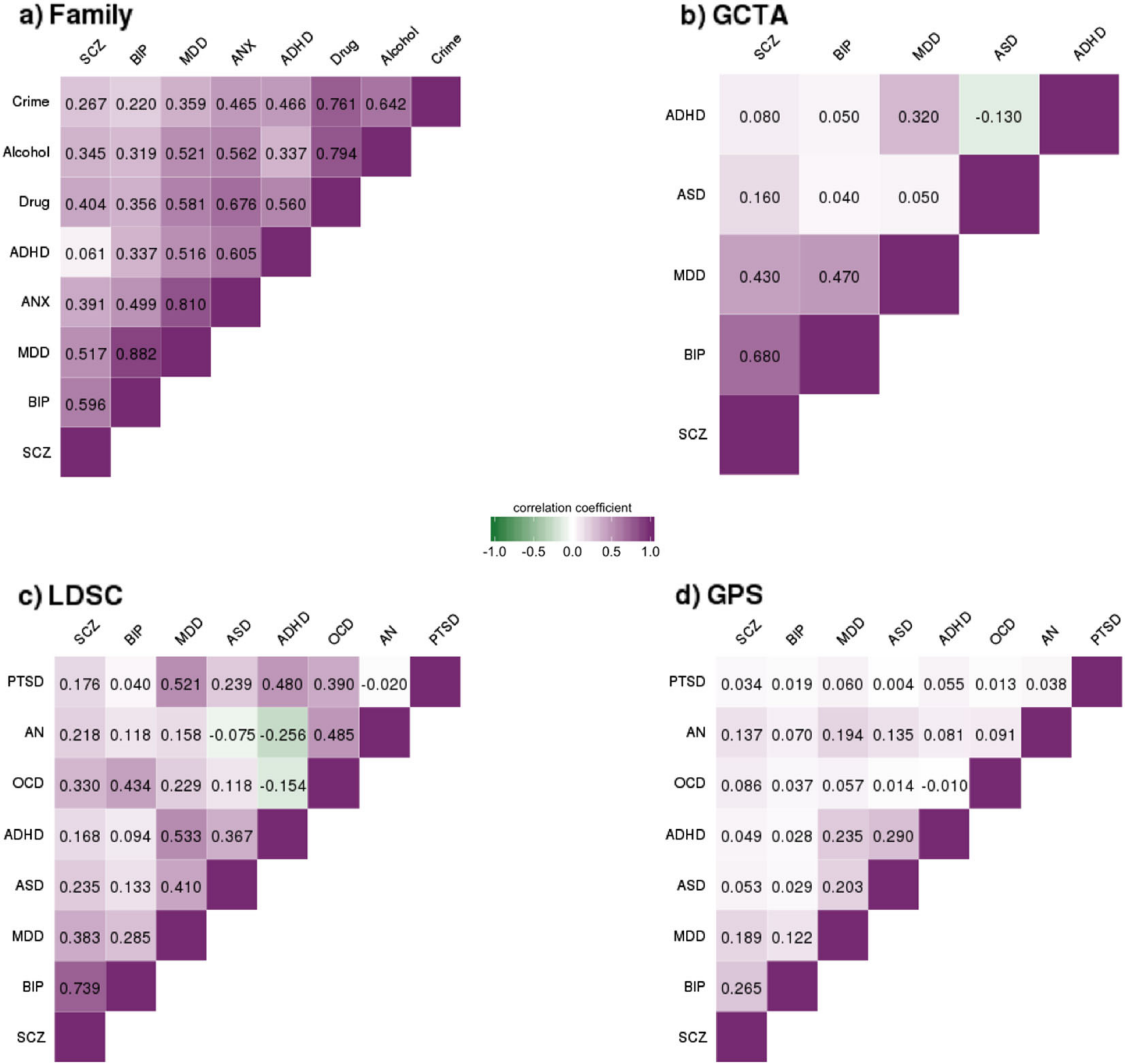
Genetic correlations from family analysis (**a**), Genome-wide Complex Trait Analysis (**b**), Linkage-Disequilibrium Score Regression (**c**) and Genome-wide Polygenic Score (**d**) analysis. Values represent genetic correlations for (**a**), (**b**) and (**c**) and Pearson’s correlation coefficients for (**d**). *SCZ* Schizophrenia, *BIP* Bipolar Disorder, *MDD* Major Depressive Disorder, *ASD* Autism Spectrum Disorder, *ADHD* Attention-Deficit/Hyperactivity Disorder, *ANX* Anxiety, *OCD* Obsessive-Compulsive Disorder, *AN* Anorexia Nervosa, *PTSD* Post-Traumatic Stress Disorder; Drug = Drug Abuse; Alcohol = Alcohol Abuse; Crime = Convictions of Violent Crimes

Figure 1 presents the genetic correlations from family analysis, GCTA and LDSC, and the correlations from GPS analysis. The average genetic correlations were 0.49 for family analysis, 0.22 for GCTA and 0.24 for LDSC, indicating general genetic overlap among psychiatric disorders. The average GPS correlation was lower (0.09), as expected. However, correlations for all four genetic approaches clustered in a strikingly similar way. Most notably, the average genetic correlations between Schizophrenia, Bipolar and Depression were consistently the largest in magnitude −0.67 for family analysis, 0.53 for GCTA, 0.47 for LDSC, and 0.19 for GPS. High genetic correlations were not driven by larger heritability estimates for these traits in comparison to the other disorders (see Supplementary Tables S2 and S3 for SNP-*h*^2^ estimates).

### Principal component analysis

**Fig. 2:**
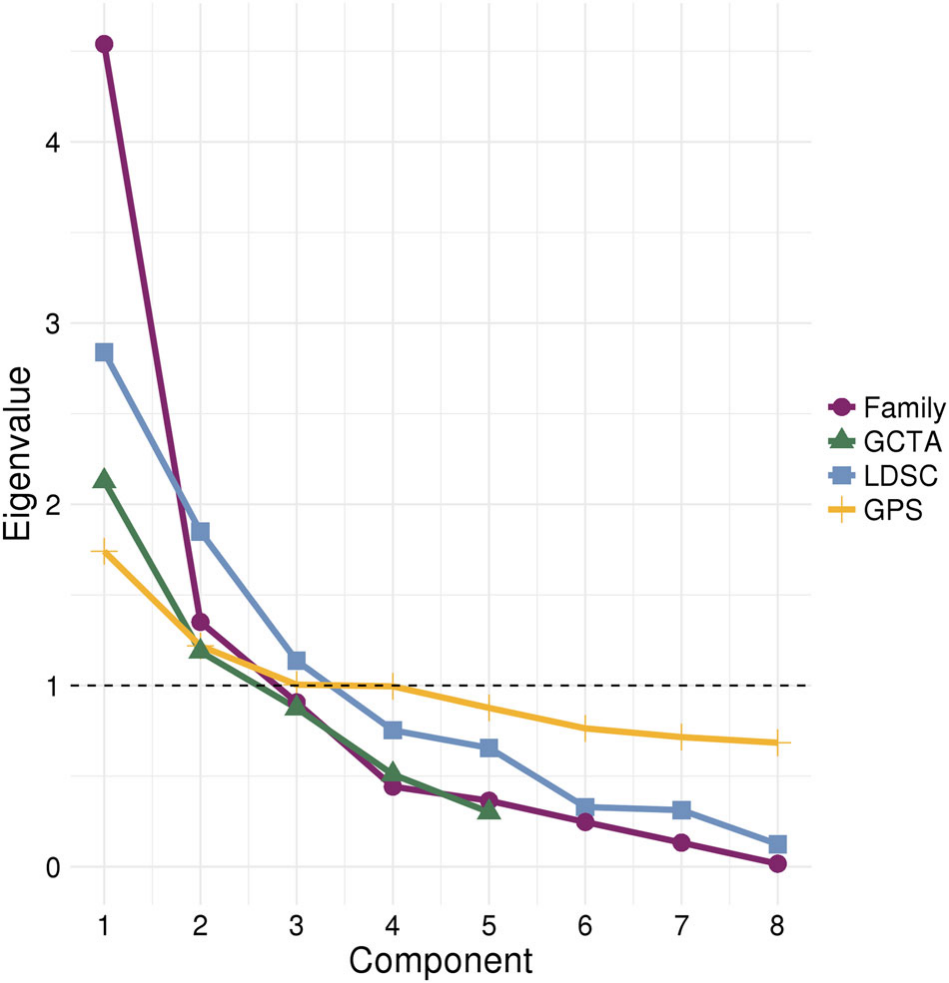
Scree plot showing eigenvalues for each principal component after performing PCA on correlation matrices for four genetically sensitive methods: family analysis, Genome-wide Complex Trait Analysis (GCTA), Linkage-Disequilibrium Score Regression (LDSC) and Genome-wide Polygenic Scoring (GPS). The dashed line represents the cut-off for principal component retention based on the Kaiser’s *λ* > 1 criterion^28^

PCA provided converging evidence for a general psychopathology factor. Figure 2 shows that all four correlation matrices yielded first unrotated principal components with larger eigenvalues than the subsequent components. The first principal component accounted for 57, 43, 35 and 22% in family, GCTA, LDSC and GPS data, respectively. (For proportion of variance explained by the other unrotated principal components, see Supplementary Table S4.)

**Fig. 3:**
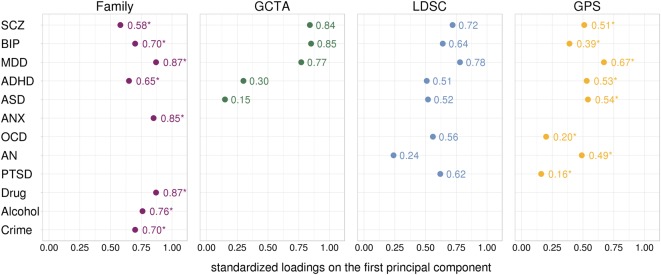
Loadings of psychopathology traits on the first unrotated principal component for each of the four types of genetic data. *GCTA* Genome-wide Complex Trait Analysis, *LDSC* Linkage-Disequilibrium Score Regression, *GPS* Genome-wide Polygenic Score, *SCZ* Schizophrenia, *BIP* Bipolar Disorder, *MDD* Major Depressive Disorder, *ASD* Autism Spectrum Disorder, *ADHD* Attention-Deficit/Hyperactivity Disorder, *ANX* Anxiety, *OCD* Obsessive-Compulsive Disorder, *AN* Anorexia Nervosa, *PTSD* Post-Traumatic Stress Disorder; Drug = Drug Abuse; Alcohol = Alcohol Abuse; Crime = Convictions of Violent Crimes. * = reached statistical significance of *p* ≤ 1.65 × 10^−41^; it was only possible to test the statistical significance for the loadings relating to GPS and family data (see Methods section for details)

Figure 3 shows first unrotated principal component loadings of all psychopathological traits for the four genetic methods. The loadings on the first unrotated principal component mirrored the genetic correlations (Fig. 1): the average loadings were 0.75 for family data, 0.58 for GCTA, 0.57 for LDSC and 0.44 for GPS. We were able to test the statistical significance of loadings in family and GPS analyses, and found that all traits significantly loaded on the first unrotated principal component (all *p*-values ≤ 1.65 × 10^−41^), even though the GPS data showed some of the lowest loadings. When we applied the conventional threshold of ≥|0.30|, we found that most of the loadings met this threshold: 100% of the disorders in family data, 80% in GCTA data, 88% in LDSC data, and 75% in GPS data. The variation in factor loadings across the four methods can be explained by the inclusion of different disorders, as average loadings for the disorders in common were highly similar (family = 0.70; GCTA = 0.69; LDSC = 0.66; GPS = 0.53).

Schizophrenia, Bipolar, and Depression consistently had the highest loadings on the first unrotated principal component across all genetic approaches with the exception of the GPS method, where Bipolar was not amongst the highest loading disorders.

### Sensitivity analyses using LDSC and GPS data

To test whether GPS results changed when applying a different prior as part of the GPS calculation, we re-ran PCA using GPS based on the fraction of causal markers of 0.10. Results were almost identical (see Supplementary Table S5).

Furthermore, it is possible that low GPS loadings were attributable to insufficient statistical power, rather than a lack of true effects. Therefore, we re-ran PCAs using LDSC and GPS data based on superceded GWA study summary statistics with smaller sample sizes, where possible (see Supplementary Table S6 for sample information). Although we found a slight reduction in the variance explained by the first principal component in LDSC data (34 vs 35%), the effect was more pronounced in the GPS data (19 vs 22%). Additionally, average GPS loadings on the first principal component decreased from 0.44 to 0.37, and only 50% of the disorder GPS met the loading threshold of ≥|0.30| . These analyses suggest that as GWA study sample sizes increase, the magnitude of factor loading effect sizes on a genetic *p* factor will approach those derived from family studies.

### Factor rotation solutions

Based on the criteria described in the Methods section, we retained two principal components for rotation for family, GCTA and GPS data, and three principal components for LDSC data (for more details, see Supplementary Table S4). However, to improve comparability of the rotated factor solutions across the four genetic methods, we kept two principal components for the LDSC data. Results of the rotation of three components for LDSC data can be found in Supplementary Table S7.

**Fig. 4:**
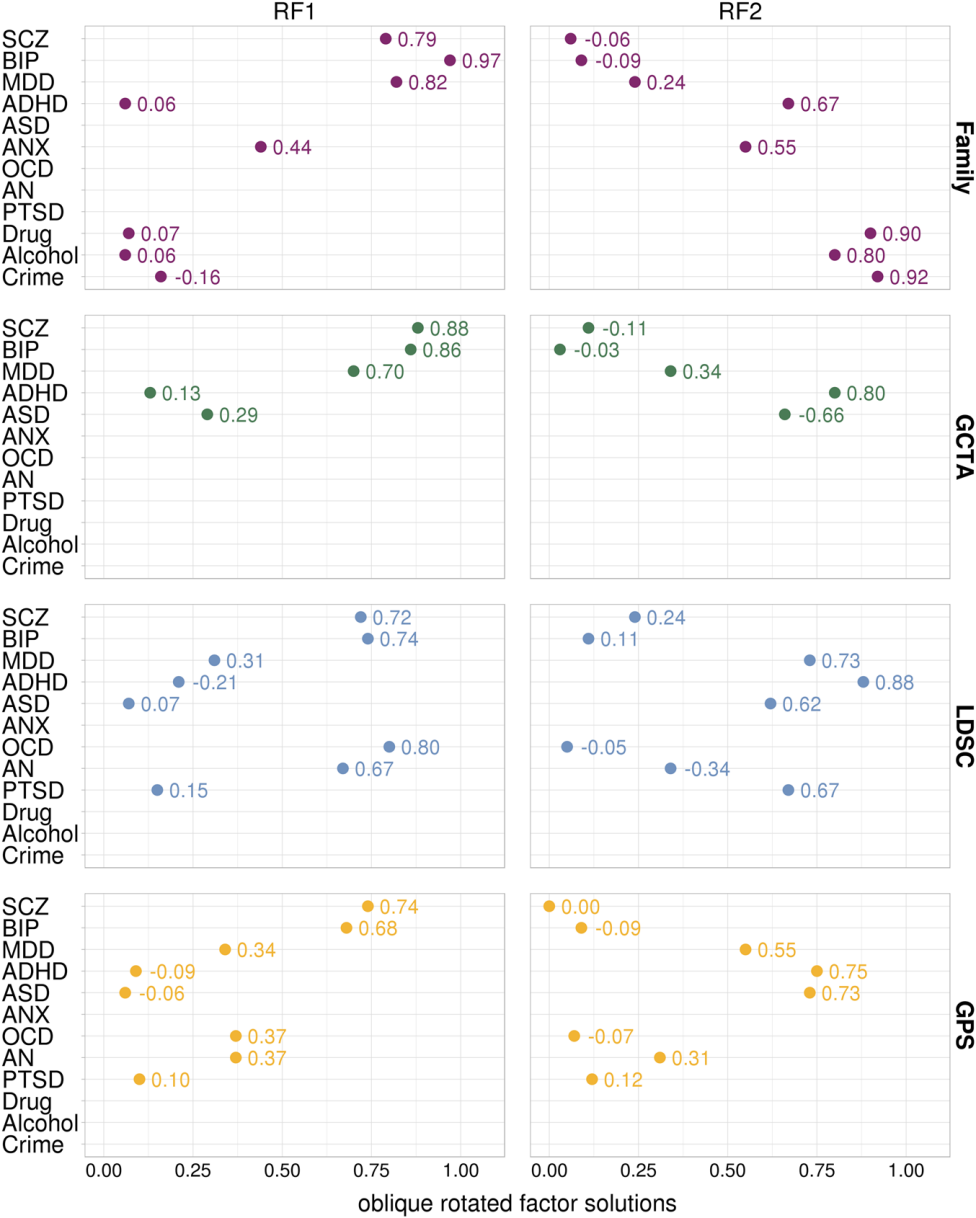
Rotated factor loadings for the four types of genetic data. *RF* rotated factor based on oblique (*Oblimin*) rotation, *GCTA* Genome-wide Complex Trait Analysis, *LDSC* Linkage-Disequilibrium Score Regression, *GPS* Genome-wide Polygenic Score, *SCZ* Schizophrenia, *BIP* Bipolar Disorder, *MDD* Major Depressive Disorder, *ASD* Autism Spectrum Disorder, *ADHD* Attention-Deficit/Hyperactivity Disorder, *ANX* Anxiety, *OCD* Obsessive-Compulsive Disorder, *AN* Anorexia Nervosa, *PTSD* Post-Traumatic Stress Disorder; Drug = Drug Abuse; Alcohol = Alcohol Abuse; Crime = Convictions of Violent Crimes

Figure 4 lists the loadings for the first two rotated factors after performing oblique rotation. Rotated factor loadings for all methods (family, GCTA, LDSC, GPS) show that Schizophrenia and Bipolar Disorder consistently load highly onto the same factor, together with Depression in the family and GCTA data. This is expected from the higher genetic intercorrelations between these traits for all methods (Fig. 1). For the remaining psychiatric traits, results were less consistent when comparing family data to genomic data (GCTA, LDSC, GPS). In part, this reflects the traits included—most notably, a Drug Abuse/Crime factor emerged from the family data because, unlike the other datasets, Drug Abuse, Alcohol Abuse and Violent Crime were included and created the first rotated factor. Anxiety also contributed to both rotated factors. For the LDSC and GPS method, which are based on the most powerful GWA studies, the second factor primarily included Depression, Attention-Deficit/Hyperactivity Disorder, Autism and Post-Traumatic Stress Disorder. Correlations between the first and second oblique rotated factors were 0.45 for family data, 0.08 for GCTA data, 0.14 for LDSC data and 0.10 for GPS data.

## Discussion

These results provide genetic support for *p*, a general factor of psychopathology that represents a single, continuous genetic dimension of the psychiatric spectrum. The four methods used to estimate genetic correlations differ substantially: quantitative genetic analysis of siblings and half-siblings^12^, GCTA estimates based on SNP differences between unrelated individuals^14^, LDSC analysis based on GWA summary statistics, and GPS for individual data presented in this paper. Nonetheless, each of the principal component analyses from the four methods yielded a general factor on which all disorders loaded, explaining between 20 and 60% of the total variance.

Schizophrenia, Bipolar and Depression are the oldest and most consistently diagnosed psychiatric disorders, yet they are consistently among the highest- loading disorders on this genetic *p* factor. This finding is unlikely to be due to some artifact of genetic analysis because it was consistent across different genetic methods applied to different samples.

It is difficult to draw general conclusions about the other disorders that varied across the four genetic methods (Obsessive Compulsive Disorder, Anorexia, and Post-Traumatic Stress Disorder, Anxiety, Drug Abuse, Alcohol Abuse and Violent Crime). However, when any of these disorders were included in a study, they consistently contributed to a genetic *p* factor in the sense that they loaded positively on the first unrotated principal component.

Although the four genetic methods yielded similar patterns of correlations and patterns of loadings on the first unrotated principal component, they differed in the magnitude of their estimates of correlations and loadings, even when only considering the disorders in common (i.e., Schizophrenia, Bipolar, Depression, Autistic Spectrum Disorder). In principle, genetic correlations calculated through GCTA and LDSC should not differ substantially from family study estimates. Even though univariate SNP-*h*^2^ is generally lower than family-*h*^2^ because the SNP-*h*^2^ estimate does not include rare variants and nonadditive effects, this downward bias influences both numerator and denominator to equal extents when calculating genetic correlations $({r}_{g}={h}_x{h}_y/{\sqrt{{h}_{{x}^{2}}}{h}_{{y}^2}})$, therefore cancelling out the bias^35^. However, if the correlation between causal SNPs is stronger for common variants than for rare variants, the SNP genetic correlation estimate would be higher than family study estimates, because only common SNPs are included in the analysis^16^. Nevertheless, for the disorders in common, family data produced higher average genetic correlations (0.49) than GCTA (0.34) and LDSC (0.37). An alternative explanation involves differences in sample ascertainment and psychiatric diagnoses. In most genomic studies, sampling strategies may select ‘pure’ cases and exclude cases with other co-occurring conditions, and such ‘pure’ cases do not represent the disordered population^36^. In contrast, family data used in this study^12^ were based on a non-hierarchical approach to classification, thus allowing for greater overlap among the disorders.

GPS results, which are based on the most conceptually distinct method, yielded the lowest overall correlations. A GPS is the aggregation of all genetic effects found in an independent GWA analysis in respect to an individual’s genotype. Therefore, GPS correlations index the extent to which the total variance of individuals’ GPS for one trait covaries with GPS for other traits. Two possible reasons why GPS correlations may be the lowest are that (i) in addition to true effects, a GPS includes the measurement error for all the SNPs tested across the genome in GWA analysis and (ii) a GPS is generated using genotypes from one cohort and effect sizes from a second, independent cohort.

What causes this genetic *p* factor? The positive manifold of the genetic *p* factor is agnostic about its causes. There are several, equally plausible hypotheses for the mechanisms that cause cross-disorder correlations^37^. One possible pathway may be *biological pleiotropy*, where DNA variants are causally involved in the development of several traits related to psychopathology. An alternative explanation is *mediated pleiotropy*, in which comorbidity occurs because DNA variants increase risk for one disorder, and then this disorder causes other disorders in turn. A third hypothesis is that DNA variants cause some general impairment that forms the core of various disorders, consequently producing genetic correlation between specific diagnoses. That is, the thousands of DNA variants associated with each symptom or disorder might affect all personality and cognitive processes that increase risk, thus providing many pathways to psychopathology.

Although it is remarkable how much genetic variance is explained by *p*, it does not explain all, or even most, of the genetic variance. Assuming a hierarchical model with *p* at the highest level^6,7^, broader psychiatric dimensions at a middle level, and specific psychopathologies at the lowest level, the question is how much genetic variance is accounted for by the three levels. In the realm of cognitive abilities, there continues to be debates about the nature of the middle level^38^.

As compared to *p*, there is less clarity in our results about the nature of the second level of the hierarchical structure, as represented by the rotated factor solutions. One rotated factor consistently includes Schizophrenia and Bipolar Disorder. However, the other rotated factor is less clear. For example, although Attention-Deficit/Hyperactivity Disorder loads on the second factor, it clusters positively with Depression and Autism Spectrum Disorder in the LDSC and GPS results, positively with Anxiety, substance abuse and Crime in the family results, and negatively with Autistic Spectrum Disorder in the GCTA and GPS results. It may be that the second level of the hierarchical structure will remain unclear until analyses of this type begin to use a transdiagnostic approach, that is, using symptoms to build a hierarchical model from the ground up. As these data become available in the future, we will be able test the genetic *p* factor model more formally by contrasting it to alternative models.

Another issue for future research is the extent to which the *p* factor is even more general than psychiatric disorders. The same approach can be used to investigate the genetic relationship between psychiatric disorders and personality traits, cognitive traits, structural and functional brain traits, medical and neurological disorders, and physiological traits. However, here we chose to focus on the extent to which a genetic *p* factor emerges from genomic analyses of psychiatric disorders themselves.

As noted, our analyses are limited to the data that currently exist, including the power of current GWA studies and the disorders included in these studies. A fundamental limitation is ‘missing heritability’, the gap between SNP-*h*^2^ and family study heritability estimates. We used the most recent publicly available GWA summary statistics, some of which are considerably underpowered. This limitation most affects our GPS analysis, which predicts genetic risk at the level of individuals. The modest SNP-*h*^2^ and measurement error of the GWA studies from which the GPS were derived are partly responsible for the low correlations between the GPS. More powerful GWA studies are in progress, and we are optimistic that new GPS will have improved predictive accuracy. More generally, GWA studies focused on phenotypic *p* should be able to capture genetic *p* to a greater extent than trying to derive genetic *p* from GWA studies of separate disorders that are sometimes diagnosed as ‘pure’ cases that exclude other diagnoses.

In conclusion, we report strong evidence for a genetic *p* factor that represents a continuous, underlying dimension of psychiatric risk using four distinct genetic methods. As GWA studies continue to increase in sample size as well as in the diversity of their target traits, our current results suggest that a genetic *p* factor could be useful in psychiatric research.

## Electronic supplementary material

Supplementary Online Material
